# New Appliances and Things Medical

**Published:** 1898-12-17

**Authors:** 


### NEW APPLIANCES AND THINGS MEDICAL.

[We shall be glad to receive, at our Office, 28 & 29, Southampton Street, Strand, London, W.C., from th manufacturers, specimens of all new preparations and appliances which may be brought out from time to time.]

### HIPI: PURE MUTTON ESSENCE.

(George Nelson Dale and Co., Limited, 14, Dowgate Hill, E.C.)

This is a good preparation of extract of mutton. It is of firmer gelatinous consistency than the usual kinds of meat preparations, and analysis of the sample submitted showed it to contain 36·2 per cent. of gelatine and proteids, including a small amount of albumen, 22 3 per cent. meat extractives, 8 3 per cent. mineral constituents, and 33 2 per cent. water. It is a concentrated extract of mutton, practically free from fat, and highly suitable as a substitute for similar beef extracts in the preparation of nutritious and appetising soups. The extract is extremely palatable, of a good pale colour, and free from all indication of having been overheated or burnt in its preparation.

### ETHEREAL ANTISEPTIC SOAP (JOHNSTON'S).

(Parke, Davis, and Co., 21, North Audley Street, London, W.)

This new liquid soap is an etherealised hydro-alcoholic solution of Castille soap. It can be readily removed by hot or cold water, will dissolve fats, and rapidly cleanse skin or other surfaces. In itself it is slightly antiseptic, but when particular antisepsis is required perchloride of mercury can be added, it is said, without decomposition or impairment of its germ destroying qualities. A liquid soap of this nature has obvious advantages for preparing the skin before operation, for cleaning the hands of surgeons before operations or after post-mortem work; and for parasitic dressing of the skin it has already many advocates, who have put its power to practical test in such cases as ringworm, scabies, &c. We understand that a solid soap is also being prepared by the same firm according to the formula of Dr. Charles McClintock, of Philadelphia, which contains 2 per cent. of mercuric iodide in combination, does not injure steel or nickel instruments, and is a most antiseptic soap for surgical and medical purposes.

### ANTISEPTIC LOZENGES.

(Cargona Company, Limited, Cross Street, Peterborough.)

Cargona lozenges are strongly antiseptic and by no means unpalatable. They are particularly recommended to the notice of nurses and others exposed to sources of infection. They may be retained in the mouth for a considerable time, and may be highly recommend to the notice of all those who are in constant attendance on infectious cases or otherwise exposed to contagion.

### BELLADONNA PLASTERS AND ANTISEPTIC LIGATURES.

(Seabury and Johnson, 31, Snow Hill, London, E.C.)

The worthlessness and unreliability of many of the belladonna plasters in the market is proverbial; many of them have been shown by independent analysis to contain none of the alkaloids of the drug. The plasters manufactured by Messrs. Seabury and Johnson may be relied upon as of standard strength, and otherwise to fulfil the conditions of a first-rate, up-to-date preparation. By an elaborate and ingenious process, the same firm are now preparing for the use of surgeons "aseptic" ligatures in small capsules. The ligatures, whether catgut or silk, are enclosed in small capsules, and, to carry out the object desired, the capsule must only be opened by the surgeon under aseptic conditions. The catgut is supplied in lengths of 30 inches, and the silk in lengths of one yard.

